# Low-temperature processing of screen-printed piezoelectric KNbO_3_ with integration onto biodegradable paper substrates

**DOI:** 10.1038/s41378-023-00489-0

**Published:** 2023-02-23

**Authors:** Morgan M. Monroe, L. Guillermo Villanueva, Danick Briand

**Affiliations:** 1 École Polytechnique Fédérale de Lausanne (EPFL), Soft Transducers Laboratory, Institute of Mechanical Engineering, 2000 Neuchâtel, Switzerland; 2 École Polytechnique Fédérale de Lausanne (EPFL), Advanced NEMS Laboratory, Institute of Mechanical Engineering, 1015 Lausanne, Switzerland

**Keywords:** Materials science, Electrical and electronic engineering

## Abstract

The development of fully solution-processed, biodegradable piezoelectrics is a critical step in the development of green electronics towards the worldwide reduction of harmful electronic waste. However, recent printing processes for piezoelectrics are hindered by the high sintering temperatures required for conventional perovskite fabrication techniques. Thus, a process was developed to manufacture lead-free printed piezoelectric devices at low temperatures to enable integration with eco-friendly substrates and electrodes. A printable ink was developed for screen printing potassium niobate (KNbO_3_) piezoelectric layers in microns of thickness at a maximum processing temperature of 120 °C with high reproducibility. Characteristic parallel plate capacitor and cantilever devices were designed and manufactured to assess the quality of this ink and evaluate its physical, dielectric, and piezoelectric characteristics; including a comparison of behaviour between conventional silicon and biodegradable paper substrates. The printed layers were 10.7–11.2 μm thick, with acceptable surface roughness values in the range of 0.4–1.1 μm. The relative permittivity of the piezoelectric layer was 29.3. The poling parameters were optimised for the piezoelectric response, with an average longitudinal piezoelectric coefficient for samples printed on paper substrates measured as *d*_33, *eff*, *paper*_ = 13.57 ± 2.84 pC/N; the largest measured value was 18.37 pC/N on paper substrates. This approach to printable biodegradable piezoelectrics opens the way forward for fully solution-processed green piezoelectric devices.

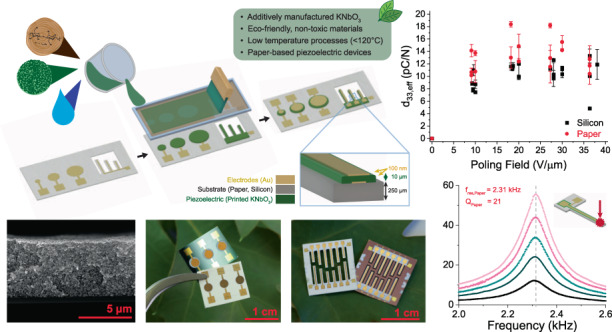

## Introduction

As global industries move further into the semiconductor era, the amount of waste generated from electronics (“e-waste”) is increasing rapidly, with an estimated worldwide production approaching 50 million megatons of e-waste annually^1–3^. Many of these electronics are difficult to recycle, containing components known to be environmentally toxic, while relying on microfabrication technologies that are energy and material intensive. It is thus imperative that alternative forms of electronics are developed using environmentally friendly processes and green materials to mitigate or reduce these negative global impacts.

To this end, a library of biodegradable materials with useful electronic properties has been identified; however, the majority of research on green device manufacture still relies on conventional microfabrication techniques^4–7^. Nevertheless, printed electronics offer an eco-friendly alternative to microfabrication in situations where scalability, cost-effectiveness and low material waste are preferred^8,9^. Combining these efforts to produce printable biodegradable electronics has found success for conductive materials (Zn, Mo, carbon) as well as inorganic (MgO and SiO_2_) and organic (Polyvinyl alcohol, cellulose and silk) insulating materials^6,7,10–12^. However, work still remains for more specialised electronic components.

Of particular note is the recent effort to transition away from environmentally harmful lead-containing materials for piezoelectric components—often as lead zirconate titanate ((Pb,Zr)TiO_3_, or PZT) and its derivatives—which are used for their high piezoelectric coefficients but now find themselves in need of replacement due to environmental concerns. While numerous Pb-free piezoelectric materials exist (e.g., BaTiO_3_, (K,Na)NbO_3_, and polymers such as poly(vinylidene fluoride-co-trifluoroethylene) abbreviated PVDF-TrFE), creating a completely green device from these materials remains a significant challenge^13–16^. An entirely green device requires a biodegradable substrate. Green substrate materials, such as paper and silk, typically have very poor temperature stabilities and are unable to withstand temperatures above 250 °C^17,18^. Any process using these substrates cannot therefore exceed this temperature, preventing the high temperature (>600 °C) sintering steps that are typically required when printing piezoelectric ceramics (as depicted in Table 1 and expanded upon in the Supplementary Information, Table SI.1).

**Table 1:** State of the art on low temperature printed piezoelectrics showing studies with the lowest reported maximum processing temperature for several commonly utilised piezoelectric materials

Material	Printing method	Substrate(s)	Electrode(s)	*T*_Max,Bulk_ (°C)	*T*_Max,Work_ (°C)	*d*_33,Bulk_ (pC/N)	*d*_33,Work_ (pC/N)	*P*_r,Work_ (μC/cm^2^)	*E*_c,Work_ (kV/cm)	Refs.
(K,Na)NbO_3_	SP	Al_2_O_3_	Pt	1060–1105	850	100–300	88	5–10	57.3	^36–39^
PZT	SP	Si	Pt, Ag	900–1200	750	593	101	–	–	^40–42^
BaTiO_3_	IJP	ZrO_2_	Pt	1100–1230	600	190	–	3.1	1.1	^43–45^
BaTiO_3_-PUA Composite	SP	Steel	Ag	–	150	–	1.31	–	–	^20^
PVDF-TrFE	IJP	PI, PET	Ag	140	140	32	–	–	–	^46,47^
ZnO	EHD	Si, PET	ITO	1100–1300	25	6–13	23.7^(PFM)^	–	–	^48–50^
KNbO_3_ (This work)	SP	Si, Paper	Au	1020–1060	120	60–110	13	–	–	^38,51^

Printing methods include SP, IJP, EHD

*SP* screen printing, *IJP* inkjet printing, *EHD* electrohydrodynamic jetting

Recent low-temperature approaches for printing piezoelectric materials involve polymer ceramic composites (typically nonbiodegradable polyvinylidene fluoride (PVDF) or polydimethylsiloxane (PDMS) embedded with ferroelectric particles)^19,20^. This approach removes the sintering step at the cost of a significant reduction in piezoelectric performance relative to bulk literature values for conventional processes^21–23^.

This work shows the first successful fabrication of fully eco-friendly KNbO_3_-based printed piezoelectric devices on green substrates^24,25^. Through careful material selection and the utilisation of printing technologies, the core challenges associated with conventional high-temperature processed Pb-based piezoelectrics are overcome. Key challenges of ink development are addressed through the adjustment of particle size and ink component compositions to develop a high-quality screen-printable ink. This study comprises the novel development of a printable KNbO_3_ ink along with the first instance of entirely low-temperature printed perovskite materials for piezoelectric applications. With a maximum processing temperature of 120 °C, this process permits the development of printed piezoelectric devices on biodegradable substrates, such as paper, for the first time. We demonstrate the versatility of this technology through the fabrication and characterisation of transducer discs and resonating structures on both silicon and paper substrates. Through poling optimisation, we attain an average piezoelectric response on paper substrates of 13.57 ± 2.84 pC/N, with maximum values reaching 18.37 pC/N. This work allows for the further development of printed green piezoelectric devices integrated with other biodegradable components.

## Methods

The development of a facile, robust process for the low-temperature printing of biodegradable KN inks consisted of three primary phases: powder processing and ink development, device design and fabrication, and characterisation. We then look to evaluate the limits of printability, the dielectric behaviour, and most critically, the piezoelectric characteristics of the KN layer as implemented in different device structures with a focus on poling optimisation. Additionally, we focus on the performance of this layer when printed on a biodegradable substrate (paper) relative to the measured performance from same process on a more conventional substrate (silicon) and the theoretical values from bulk KN properties.

**Fig. 1:**
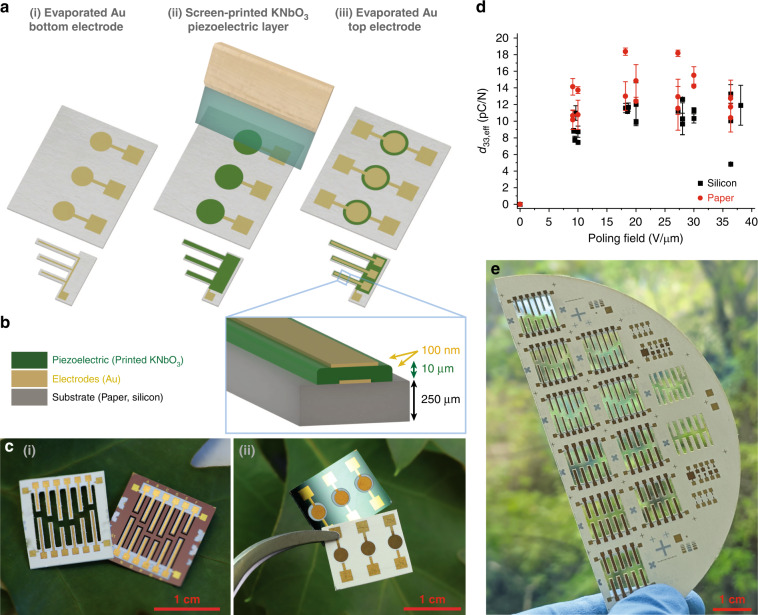
Overview of this work. **a** Simplified process flow for the fabrication of printed piezoelectric devices including (i) bottom electrode deposition via thermal evaporation, (ii) screen printing of the KNbO_3_ piezoelectric layer and (iii) top electrode deposition via thermal evaporation. **b** Cross-section of the printed device layers indicating layer thicknesses. **c** Images of the as-fabricated (i) cantilever and (ii) capacitor structures printed on both paper and silicon substrates. **d** Effective *d*_33_ response achieved as a function of the poling field for capacitor structures, measured via the Berlincourt method. **e** Cantilever devices printed at the wafer scale on paper substrates

Two primary device architectures were fabricated, parallel plate capacitors and resonant cantilevers, each produced on both silicon and paper substrates. This combination facilitates a comparison of device behaviour on different substrates, a proof of concept for versatile printing capabilities, and a characterisation of piezoelectric behaviour in both longitudinal and transverse modes. Figure 1 shows a summary of the device architectures, fabrication, scalability and key piezoelectric response of the as-fabricated devices, all of which are discussed in further detail. Figure 1a shows a simplified fabrication process for these devices, with a cross-section of the finished device stack shown in Fig. 1b, including approximate layer dimensions. The finalised printed devices for both the parallel plate capacitor and cantilever structures are imaged in Fig. 1c.

Crucial to this process, all component materials selected for these devices are nontoxic: the ink (composed of KN particles, ethyl cellulose and 1-pentanol), the electrodes (composed predominantly of gold, which is an inert noble metal), and the paper substrate (primarily composed of cellulose).

### Powder processing and ink development

Initial steps towards ink development focused on selecting suitable eco-friendly component materials for ink composition and printability. As discussed previously, potassium niobate was selected as the active piezoelectric ingredient. The remaining ink components (a binding agent and carrier fluid) were then carefully chosen to develop an ink that was highly stable, reproducible and screen-printable. The ink should have a viscosity in the range of 1–10 Pa.s and produce printed layers in the range of 5–20 μm.

To select a binding agent, it was necessary to select a material that could sufficiently adhere the active ingredient to a substrate, withstand the ink drying process temperatures, and ideally interact minimally with water (and thus reduce the system’s sensitivity to humidity). To this end, the cellulose derivative ethyl cellulose (EC)—a polymer biodegradable in the presence of a wide range of microbial and fungal organisms—was selected^26,27^. A decomposition temperature of >150 °C and insolubility in water made ethyl cellulose an excellent choice as a binding agent^28^.

The carrier fluid of a screen-printable ink must take into consideration, among other properties, fluid volatility, viscosity and interactions with active ingredients. Therefore, highly volatile solvents, including 1-propanol and acetone, were deemed unsuitable for screen printing applications. Despite being the optimal choice for a biodegradable fluid, the high surface tension of water made particulate dispersions unstable without the addition of complex surfactants. Instead, a low-volatility alcohol, 1-pentanol (C_5_H_11_OH), was selected. This solvent, well known for its use in printing applications due to its low volatility, has been the solvent of choice for many screen-printable perovskite inks and can be produced sustainably via biofermentation^29,30^.

With materials selected, the focus turned to identifying an ink composition to produce the desired ink properties. Several ink compositions composed of 50–80 wt.% KNbO_3_ active ingredient (99.999% purity, purchased from Alfa Aesar), 5–20 wt.% ethyl cellulose binding agent, and 10–40 wt.% 1-pentanol carrier fluid were evaluated and then iterated successively to define a narrow range of compositions suitable for the targeted printing specifications.

To ensure high-quality screen-printing in the targeted thickness range of 5–20 μm, the constituent active ingredient particles must requisitely be on the submicron scale in diameter. However, analysis conducted during the initial ink development phase determined the average particle size of the as-received KN powder to be in the range of 1–10 μm in diameter (see Fig. SI.2.1) and thus nonideal for the intended application. Preprocessing was therefore deemed necessary to reduce the average KNbO_3_ particle diameter, conducted via ball milling to achieve an appropriate particle size for mixing into a screen-printable ink (see “Materials and methods” section for further details).

The influence of particle size on piezoelectric response in ferroelectric materials has been well-documented, with optimum results typically in the range of 1–10 μm average diameter and dependent heavily on the specific ferroelectric material being studied^31–34^. Thus, in reducing the particle size to increase the quality of printed layers, the resulting material piezoelectric response was reduced as well. However, due to the limitations of this printing technique, evaluation of this influence has yet to be fully explored.

**Fig. 2:**
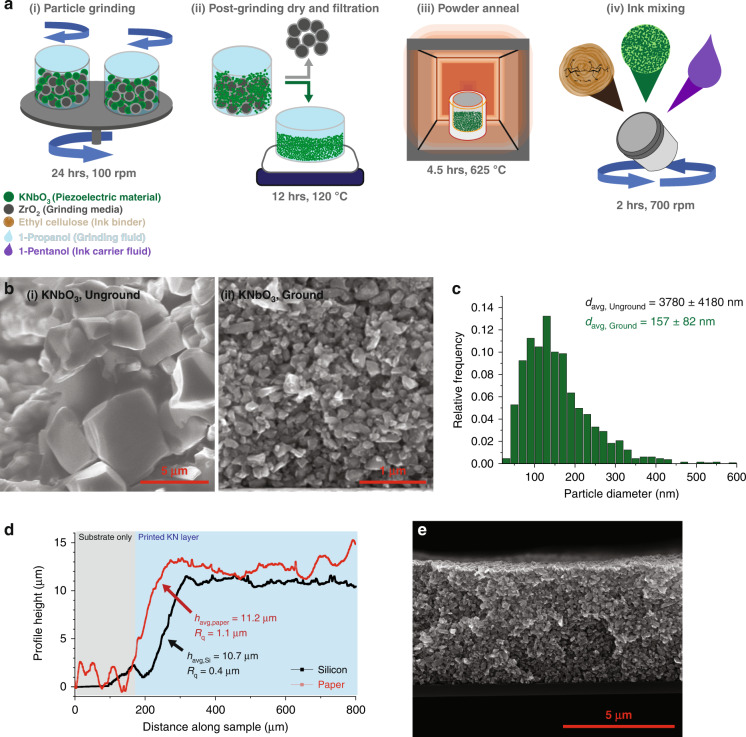
Summary of the powder grinding process. **a** Powder process diagram including (i) grinding of KN particles, (ii) separation of ground particles from grinding media and drying off of 1-propanol grinding aid, (iii) annealing of powder to improve particle crystallinity and (iv) mixing of screen-printable KN ink. **b** SEM images of (i) unground and (ii) ground KN particles, **c** Particle size distribution of ground KN material, and **d** Edge surface profile of KN ink as-printed on paper and silicon substrates noting layer thickness and surface roughness. **e** SEM cross-sectional image of KN ink printed on silicon substrate

Schematics of the powder milling and ink development processes are detailed in Fig. 2a, including steps of (i) grinding the KN particles in a planetary ball mill, (ii) separating the ground particles from the milling media and solvent, (iii) reannealing the particles to improve crystallinity, and (iv) mixing the ink.

To achieve a homogeneous distribution of particulates, 5 g of KNbO_3_ powder was mixed with 10 mL of 1-propanol and 125 g of ZrO_2_ grinding media (2 mm Ø); the system was placed in a planetary ball mill for 24 hrs, with a directional switching period of 0.3 h and a jar rotational speed of 200 rpm (Fig. 2ai). Next, the powder-solvent slurry was separated from the grinding media and placed on a hotplate at 120 °C for 12 h to evaporate the solvent, resulting in a dry, ground powder (Fig. 2aii). Finally, the resulting powder was annealed to improve particle crystallinity. Annealing was conducted in a muffle furnace at 625 °C for 4.5 h, and was followed by a passive cooling step of ~16 h (Fig. 2aiii).

The particle size distribution for ground KNbO_3_ particles was determined via image analysis using scanning electron microscopy (SEM) and image processing software (Fig. 2b). Image analysis showed a significant reduction in size from the initial, unground KN powder (Fig. 2bi) to the finalised, ground KN powder (Fig. 2bii). The postgrinding particle size distribution is shown in Fig. 2c, showing a reduction in the average particle diameter of the KN particles from 3.78 ± 4.18 μm in the unground powder to 157 ± 82 nm in the ground powder. Details of the particle size distribution analysis could be found in SI.2.

After grinding, further iterations were required to optimise the ink composition due to the influence of particle size on ink rheology. The binder content was constrained to a range between 5–10 wt.%, as binder content higher than 10 wt.% resulted in printed layer delamination; while binder content below 5 wt.% compromised film integrity. The carrier fluid content was refined to the range of 30–40 wt.%, as fluid content over 40 wt.% reduced ink viscosity to a value outside of the workable range of screen printing and fluid content of <30 wt.% resulting in poorly dispersed, nonhomogeneous inks. The finalised ink was achieved by mixing the three components in a proportional ratio of 58:7.5:35 wt.% KN:EC:1-pentanol (Fig. 2aiv). The mixture was mixed in a planetary mixer with 5 g of Al_2_O_3_ milling media at 700 rpm for a total of 2 hrs in 30 min intervals to reduce solvent evaporation due to system heating. Slight additions of 1-pentanol were required to adjust for the mass lost to evaporation as needed to attain the targeted ink viscosity. Once mixed, ink was stored at 5 °C in a well-sealed container. This ink was highly stable and viable for printing after more than 6 months under these storage conditions.

Upon printing, the surface profilometry of the KN layers (Fig. 2d) showed average thicknesses of 10.7 μm on silicon and 11.2 μm on paper. The surface roughness of the printed KN layers was slightly greater when printed on paper relative to when printed on silicon; attributed to the initial roughness of the paper itself, with an RMS layer roughness of 0.4 μm on silicon substrates and 1.1 μm on paper substrates. However, this surface roughness was determined to be acceptable for device fabrication, as shown in SEM imaging in Fig. 2e.

### Device design

Two architectures were selected to evaluate the printed KN material and demonstrate its applicability in piezoelectric devices. Both designs are based on a standard parallel plate capacitor construction, with a planar piezoelectric layer between the top and bottom electrodes. The first design involves parallel plate capacitors with circular footprints of varied surface areas *A* = 1, 5, 10, and 20 mm^2^. The direct piezoelectric effect could then be utilised to determine the effective longitudinal piezoelectric coefficient, *d*_33,*eff*_, from these devices via the Berlincourt method.

Additionally, this design permitted the determination of the printed piezoelectric layer relative permittivity on each substrate according to a parallel plate capacitor assumption as follows:

1 $$C = \left( {\frac{A}{{h_{piezo}}}} \right)\varepsilon _r\varepsilon _0$$

where *C* is the measured device capacitance, *A* is the surface area of the printed capacitor, *h*_*piezo*_ is the thickness of the printed layer, *ε*_0_ is the vacuum permittivity$\left( {\varepsilon _0 = 8.854\frac{{pF}}{m}} \right)$, and *ε*_*r*_ is the relative permittivity of the printed layer.

To accompany the circular capacitor structures, a second design was proposed for the fabrication of piezoelectric cantilevers. By taking advantage of the indirect piezoelectric effect and analysing the characteristics of the piezoelectric cantilevers at resonance, the effective transverse piezoelectric coefficient, *d*_31,*eff*_, could be determined. With the assumption that the thermally evaporated metallic electrodes do not contribute significantly to the system, the cantilever could be approximated as a bimorph composed of a substrate and a piezoelectric layer such that the resonant frequency is predicted as follows:

2 $$f_r = \frac{{1.875^2}}{{2\pi L^2}}\sqrt {\frac{{\left\langle {EI} \right\rangle _{eq}}}{{\left\langle {\rho A} \right\rangle }}}$$

where *f*_*r*_ is the resonant frequency of the cantilever, *L* is the length of the cantilever, 〈*EI*〉_*eq*_ is the flexural rigidity of the beam, and 〈*ρA*〉 is the mass per unit length of the beam.

This predictive model was used in tandem with known fabrication limitations and design constraints to identify physical device parameters that simultaneously enable the reproducible fabrication and quality characterisation of the resulting cantilevers. Due to their novelty for use in this application, several material properties for KN and paper were estimated for this model; in particular, the densities and Young’s moduli of the paper substrate and ink printed layers were initially estimated. These values were updated accordingly as new information was gained empirically through successive development iterations. A comparison of this model to the built devices is discussed later. The specific model parameters and design constraints for this system are found in SI.3.

Based on this model, device dimensions were selected that would allow for the fabrication of cantilevers on both silicon and paper substrates of the same dimensions that resonate in the target range of 10^4^–10^5^ Hz (permitting high-quality characterisation with available instrumentation). All designed cantilevers had a width of 700 μm, thickness of 200 μm and lengths of 3.1, 4.1 or 5.1 mm (see SI.4 for further details).

### Device fabrication

**Fig. 3:**
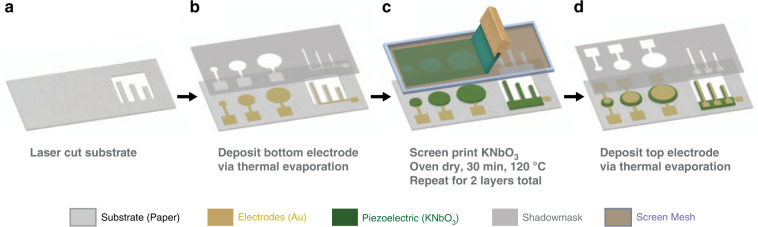
Full process flow diagram for piezoelectric device fabrication. **a** Precutting the substrate for cantilever devices using laser cutting, **b** deposition of the Au bottom electrode via thermal evaporation, **c** screen printing of the KNbO_3_ ink repeated once, with a 30-min oven drying step at 120 °C after each layer and **d** deposition of the Au top electrode via thermal evaporation

Specialty printing paper for use with printed electronics was utilised as a substrate due to its low surface roughness (~2–3 μm) and robust thermal properties (stable up to 200 °C) relative to other biodegradable substrates. The full fabrication process is depicted in Fig. 3 for the structures made on paper substrates. For the silicon reference samples, standard microprocessing techniques were utilised instead, and a description of the process is in the “Materials and methods” section. For the samples on paper, cantilever shapes were first laser cut from the substrate (Fig. 3a). Next, Cr/Au bottom electrodes were deposited for both piezoelectric structures via thermal evaporation (Fig. 3b). A manual screen printer was then utilised for the printing of piezoelectric layers. The piezoelectric KNbO_3_ ink was printed on all substrate and device iterations in 2 layers, with a 30 min drying step at 120 °C in an oven after printing each layer (Fig. 3c). Printing two layers of piezoelectric significantly improved the device yield because the increased layer thickness compensated for the initial surface roughness of the paper substrate, thereby reducing spurious shorting across electrodes. Once the printing of the piezoelectric layer was complete, top gold electrodes were deposited on all samples via thermal evaporation using the same process as for the bottom electrodes (Fig. 3d).

## Results and discussion

### Ink and printed layer properties

A summary of the ink and device parameters is shown in Table 2. The average KNbO_3_ particle diameter after grinding was 157 ± 82 nm (Fig. 2c). The ink density was 3.17 ± 0.11 g/cm^3^, closely matching the predicted value of 3.22 g/cm^3^. The ink viscosity was 6.14 Pa.s at a shear rate of 100 s^−1^. Ink adhesion was consistent across substrate materials, with an ASTM F1842–15 rating of 4B-5B, showing good adhesion to the substrates. Further measurement information regarding density measurements, ink rheology and layer adhesion is detailed in the SI (sections SI.5–SI.7, respectively).

**Table 2:** Key parameters of printed piezoelectric devices as compared to reference values

Property	Units	Value	Std dev.	Value	Std dev.	Lit. value (bulk)	Refs.
		On silicon		On paper			
Density	g/cm^3^	3.17	0.11	–	–	4.37	^52^
Adhesion	[−]	4B-5B	–	4B-5B	–	–	–
Layer thickness	μm	10.7	0.16	11.2	0.15	–	–
Surface roughness	μm	0.4	0.14	1.1	0.28	–	–
Relative permittivity	[−]	29.5	0.2	29.3	0.1	35.14	^53^
*d*_33,*eff*_	pC/N	9.96	1.67	13.57	2.84	60–110	^51^

The initial surface roughness of the silicon and paper substrates was 2.3 ± 0.5 μm for paper and <5 nm for silicon. Figure 2(d) shows a representative edge profile of printed layers on both paper and silicon substrates; Fig. 2(e) shows an SEM image of the KNbO_3_ layer in the cross-section as printed on silicon. The KN layer thickness of printed devices was 11.2 ± 0.2 μm for capacitor structures on paper, while it was 10.7 ± 0.2 μm for samples on silicon, scaling well relative to the single-layer thickness of 5.5 μm. The RMS surface roughness of the printed layers was 1.1 ± 0.3 μm for the structures on paper and 0.4 ± 0.1 μm for those on silicon, resulting predominantly from the greater initial surface roughness of the paper substrate.

With regard to the fabrication process, printing was conducted on a wafer-sized area of 100 × 100 mm^2^, with a device failure rate of <10% in all cases (defined by the number of damaged or shorted devices upon completion of the fabrication process). This finding demonstrated the robustness of the process, despite the tight alignment tolerance of 100 μm for cantilever devices. The smallest printed features were 250 μm in width with a standard deviation of 15 μm (~5%). This result matched well with the recent limits to line resolution in screen printing applications^8^, which are generally 50–100 μm.

**Fig. 4:**
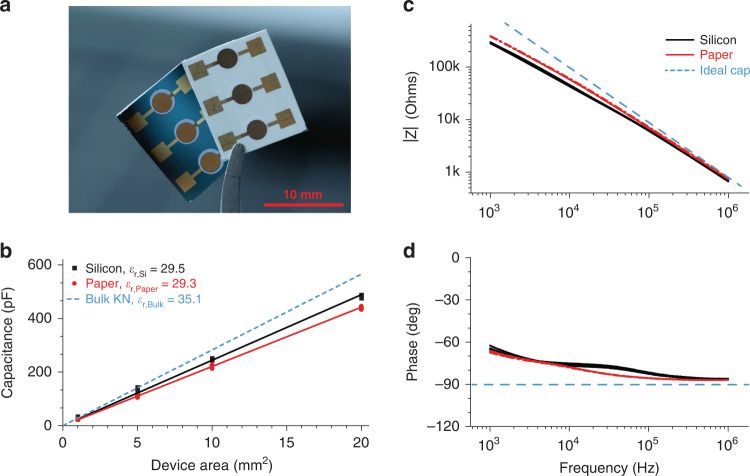
Dielectric characterization of fabricated devices. **a** Close-up image of the printed circular capacitor structures, showing those with surface areas of 5 mm^2^. **b** Capacitance versus device area trends for capacitors on both substrates annotated with noted relative permittivities, as calculated at 100 kHz, with comparison to bulk KN behaviour. **c** Impedance and **d** phase spectra of characteristic printed piezoelectric cantilevers on both silicon and paper substrates with comparison to an ideal capacitor of the same parameters

Impedance data was collected using the circular capacitor structures on both paper and silicon substrates (circular capacitor devices are shown in Fig. 4a with 5 mm^2^ surface areas). Impedance spectroscopy was used to characterise the dielectric properties of the as-fabricated capacitors in the range of 10^3^–10^6^ Hz. Figure 4b shows a comparison of capacitance versus the device surface area for samples on both paper and silicon substrates, with the calculated relative permittivity values annotated for samples on the two substrates relative to bulk reference values.

The relative permittivity was calculated at 100 kHz to reduce the influence of ambient humidity on measurements. The calculated relative permittivity of KN capacitors on paper was *ε*_*r, paper*_ = 29.3 ± 0.1 relative to those on silicon *ε*_*r*, *Si*_ = 29.5 ± 0.2, showing agreement across substrates, supporting the argument that substrate material did not significantly influence dielectric behaviour. Both values were below the bulk relative permittivity, *ε*_*r, Bulk*_ = 35.1, due to the presence of ethyl cellulose (*ε*_*r*, *EC*_ ≈ 2.8–3.9) in the composited ink layer^35^. Exemplary impedance spectra showing absolute impedance and phase data for circular capacitors with a surface area of 10 mm^2^ on both silicon and paper substrates are shown in Fig. 4c. The impedance spectra for these devices deviated from ideal capacitor behaviour, indicating that there was some electrical leakage present in the capacitor system.

### Poling optimisation and piezoelectric properties

**Fig. 5:**
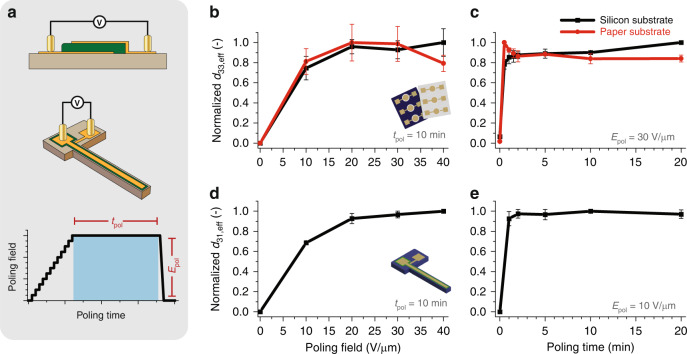
Optimisation of the poling parameters for piezoelectric coefficients *d*_33,eff_ and *d*_31,eff_. All values are reported as the normalised piezoelectric response. **a** Schematic of devices used for poling optimisation and applied poling ramp. **b** Optimisation of *d*_33,eff_ as a function of poling field as measured from printed circular capacitor devices via the Berlincourt method. **c** Optimisation of *d*_33,eff_ as a function of poling time as measured from printed circular capacitor devices via laser Doppler vibrometry. **d** Optimisation of *d*_31,eff_ as a function of poling field as measured from printed cantilever devices via laser Doppler vibrometry. **e** Optimisation of *d*_31,eff_ as a function of poling time as measured from printed cantilever devices via laser Doppler vibrometry

The piezoelectric response, as both *d*_33,*eff*_ and *d*_31,*eff*_, was optimised as a function of the poling field and soak time to determine the ideal poling parameters. The direct piezoelectric effect was used to determine *d*_33,*eff*_ values via the Berlincourt technique (see SI.8 for details of the setup and methodology used); the indirect piezoelectric effect was evaluated using laser Doppler vibrometry (LDV) to further support those measurements. The results of this optimisation study are detailed in Fig. 5, wherein trends are normalised to the maximum measured values for each test and substrate. The indirect piezoelectric effect was used to determine *d*_31,*eff*_ from cantilever devices in resonance using LDV, as detailed below.

Devices were poled at room temperature via direct (contact) poling using the deposited electrodes. A stepped voltage ramp (Fig. 5a) was implemented to attain the setpoint for poling using a programmable high voltage power supply to control the voltage ramp (and thus, the applied poling field). The target poling field, *E*_*pol*_, was maintained for a set time period, *t*_*pol*_; upon completion of the soaking period, the voltage was dropped to 0 V/μm in a single step. The applied electric fields utilised in this poling optimisation were 0, 10, 20, 30 or 40 V/μm with ramp steps equivalent to 1 V/μm every 5 s, and the poling times were 0, 0.5, 1, 2, 5 10, or 20 min. The maximum applied electric field was limited to 40 V/μm due to dielectric breakdown.

The optimisation according to the applied poling field (Fig. 5b, d) showed that poling saturation was attained at applied fields of 20 V/μm and higher, after which the measured piezoelectric response was within 20% of the maximum. A similar trend was found for poling time optimisation (Fig. 5c, e). After 0.5 min of poling, the measured *d*_33,*eff*_ and *d*_31,*eff*_ values were within 20% of the maximum measured value. From this, it was determined that ideal poling conditions occurred at poling fields between 20 and 40 V/μm and poling times >0.5 min. For further studies, 30 V/μm and 5 min were selected to mitigate the risks of dielectric breakdown and reduce the poling duration.

In terms of absolute measurements, the average piezoelectric coefficient for samples printed on paper substrates was *d*_33, *eff*, *paper*_ = 13.57 ± 2.84 pC/N as compared to those printed on silicon, *d*_33,*eff*, *Si*_ = 9.96 ± 1.67 pC/N (Fig. 1d). The highest attained *d*_33,*eff*_ was 18.37 pC/N on paper substrates and 13.28 pC/N on silicon substrates. The variance in piezoelectric response was most likely attributed to interactions of the piezoelectric layer with the two different substrates, with more clamping in samples on stiff silicon substrates as opposed to the paper substrates. Poling optimisation trends were validated using LDV measurements.

**Fig. 6:**
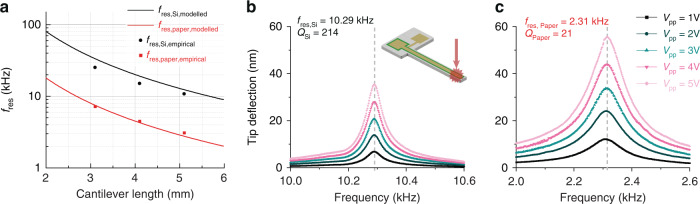
Resonance behavior of fabricated cantilever devices. **a** Modelled and empirical values of cantilever resonance frequencies on both silicon and paper substrates. **b** Silicon and **c** paper cantilever tip displacements at resonance as functions of frequency and actuating voltage, with annotated resonant frequencies and quality factors for samples processed and poled under the same conditions

We targeted the characterisation of the piezoelectric coefficient transversal to the electric field, i.e., *d*_31,*eff*_. To do so, we characterised the out-of-plane movements of cantilever beams (consisting of Si or paper, metal electrodes, and printed KN) using LDV to quantify the displacement around the first out-of-plane mode of each cantilever. As depicted in Fig. 6a, the empirical resonance frequencies of the fabricated devices matched well with the predicted values as modelled in the design phase. The small perturbations were attributed to deviations in design configurations during fabrication, along with small variances in the printed layer properties.

Cantilever devices on both substrates were then poled under a set of applied electric fields and poling times (following the values mentioned previously). In actuating each cantilever with a signal of known amplitude (1–5 V_pp_), the vertical displacement at the cantilever tip was collected over the frequency range surrounding resonance. Thanks to a Lorentzian fit, it was possible to decouple the amplification due to the quality factor from the actual piezoelectric efficiency, and thus, *d*_31,*eff*_ was extracted (see SI.9 for further details on the analysis methodology). Figure 6b, c show two typical results on Si and paper cantilevers, respectively. We note the wider peak of the paper sample, which we attributed to the large material losses that can be found with paper substrates relative to silicon. This finding was true for all poling conditions and cantilever lengths, with an average quality factor of cantilevers on silicon of 330 ± 110 and on paper of 25 ± 1.

These measurements showed that the dependence of the cantilever actuation efficiency (proportional to *d*_31,*eff*_) on the poling conditions followed the same trend as that for *d*_33,*eff*_. However, the actual extracted values of *d*_31,*eff*_ did not correspond to what was expected from the *d*_33,*eff*_ values. For samples on silicon, *d*_31,*eff,Si*_ was 0.11 ± 0.03 pC/N, while on paper, *d*_*31,eff,Paper*_ was 0.12 ± 0.02 pC/N. This result constituted a difference approaching three orders of magnitude between *d*_33,*eff*_ and *d*_31,*eff*_ on both substrates. We believe that these low *d*_31,*eff*_ values stem from suboptimal adhesion between the KN printed layer and the underlying materials. To generate out-of-plane movement in a cantilever structure, the piezoelectric layer must generate a bending moment. To do so, the expansion of the piezoelectric layer must be conveyed to the rest of the structure, for which a significant shear stress must be generated. If the adhesion was not large enough to sustain said stress, the quantification of *d*_31,*eff*_ with our method was flawed; however, we could still draw qualitative conclusions on the dependence on poling conditions. This result requires further study into the mechanical properties of the layer before enhancements could be made.

## Conclusions

A process was realised to manufacture eco-friendly printed piezoelectric devices at low temperatures, enabling their integration with temperature-sensitive substrates and targeting the development of fully biodegradable printed piezoelectrics. A screen-printable ink was developed for printing potassium niobate powder, and then devices were designed and manufactured to characterise this ink and evaluate its applicability as a printed piezoelectric material. Devices were printed with high reproducibility on both silicon and paper substrates at a maximum processing temperature of 120 °C. The average measured piezoelectric coefficient for samples printed on paper substrates was *d*_33,*eff,paper*_ = 13.57 ± 2.84 pC/N, with the largest measured value of 18.37 pC/N on paper substrates. The piezoelectric behaviour of the devices showed a reduction relative to bulk coefficients for KN; however, the response was still of a value applicable for integration into devices, and further studies could be conducted to optimise ink composition towards this end. Future works would aim to further develop this process on two axes. First, ink properties would be tuned to improve transverse piezoelectric behaviour. Additionally, this process would be fully integrated with printed biodegradable conductors to produce fully solution-processed, green piezoelectric devices.

## Materials and methods

### Materials

Substrates utilised included silicon wafers (100 mm Ø, 525 μm thickness, p-type, resistivity 0.1–100 Ω/cm^2^) with a surface coating consisting of 500 nm wet thermal silicon oxide and ArjoWiggins PowerCoat XD 200 screen printing paper with a thickness of 200 μm.

The ink components included KNbO_3_ powder (CAS 12030-85-2, Alfa Aesar Puratonic 99.999% (metal basis)), ethyl cellulose (CAS 9004-57-3, Merck, viscosity 10 cP, 5% in toluene/ethanol 80:20 (lit.), extent of labelling: 48% ethoxyl) and 1-pentanol (CAS 71-41-0, Merck, ReagentPlus, ≥99%).

### Manufacturing

A QM-3SP2 planetary ball mill was utilised for particle grinding. The milling media used for particle grinding was ZrO_2_ milling media, 2 mm Ø (Retsch Product # 05.368.0089). The Teflon milling vessel (72 mL volume) was fabricated in-house. The oven used for particle annealing was a Thermo Fischer Thermolyne F6020-33-80 Tabletop muffle furnace. Ink mixing was conducted in a Thinky ARE-250 planetary mixer using 5 g of 5 mm Ø Al_2_O_3_ spherical milling media (Retsch Product # 05.368.0019).

Paper substrates were pre-cut via laser cutting using a Trotec Speedy 300 (60 W) laser cutter to match the dimensions of a silicon substrate to enable a consistent process across all samples. The cantilevers were pre-cut from the paper substrates. No pretreatments were required on paper substrates prior to further processing, as opposed to silicon, for which surface treatment was necessary for electrode adhesion. Silicon substrates were pretreated using an O_2_ plasma cleaned (Diener ATTO, 100% Power, 10 min). For all samples, both the bottom and top electrodes consisted of a 10 nm Cr adhesion layer followed consecutively by a 100 nm layer of Au.

For exclusively the cantilever samples processed on silicon, the process for bottom electrode deposition and cantilever release deviated slightly. First, the Cr/Au bottom electrode was patterned in a cleanroom using a lift-off process. Once complete, the cantilever structures were partially etched from the top using deep reactive ion etching (DRIE) and then released fully by mechanically thinning the wafer from the backside. This action reduced the thickness of the wafer from 525 to 247 μm. Once the cantilevers were released and the gold bottom electrodes were deposited on both substrate materials, the process became the same as that for the other samples for the remaining fabrication steps.

A manual screen printer (Charmhigh 3040 High Precision Manual Solder Paste Printer) was used for printing piezoelectric ink, with a maintained substrate–mesh gap of 2 mm. Mesh fabrication was provided by Serilith AG (Balwil, CH) using polymer mesh (PME 120-30Y) and steel mesh (SD 40/25) materials. The ink layers were dried in a Memmert UF110plus oven at 120 °C for 30 min after each layer. A programmable high voltage power supply (Peta-Pico-Voltron, developed in-house) was used for direct poling of devices.

### Characterisation

Ink properties were analysed using a Keyence VHX Digital Microscope Series optical microscope, a JEOL JSM-7500TFE scanning electron microscope, and a TA Instruments DHR-2 Rotational Rheometer, with particle size distribution analysis conducted using ImageJ processing software. Adhesion measurements were characterised in accordance with ASTM F1842–15.

The device properties were characterised using an Ambios XP-2 profilometer. The dielectric properties were measured using a Digilent Discovery 2 (SKU 410-321) all-in-one test and measurement device and an Agilent 4294A impedance analyser. Laser Doppler vibrometry was conducted using a PolyTec MSV-400 microscope scanning vibrometer utilising a VD-06 velocity decoder. Direct piezoelectric effect measurements were measured using an in-house Berlincourt meter courtesy of Dragan Damjanovic. The metre showed a noise threshold *d*_33,*eff*_ of ~0.05 pC/N, for which all measurements were appropriately compensated.

## Supplementary information

Supplementary Information
