# Supplementary material for: Low-temperature processing of screen-printed piezoelectric KNbO3 with integration onto biodegradable paper substrates
Source: Microsyst Nanoeng. 2023 Feb 23;9:19. doi: 10.1038/s41378-023-00489-0 (PMC9946825; doi:10.1038/s41378-023-00489-0)
Supplement: Supplementary file 1 — Supplementary Information [file 41378_2023_489_MOESM1_ESM.docx]

Supplementary Information for Low temperature processing of screen printed piezoelectric KNbO_3_ with integration onto biodegradable paper substrates

**M. Monroe, L. G. Villanueva, D. Briand**

# SI.1. State of the Art for 2D printed Piezoelectrics

**Table SI.1**. Expanded state of the art on 2D printed piezoelectrics, including information regarding printing method, substrate and electrode materials, processing temperatures, and piezoelectric response characterization. Abbreviations for printing methods include Screen Printing (SP); Digital Light Processing (DLP); Inkjet Printing (IJP); Electrohydrodynamic Jetting (EHD).

| Author | Year | Piezo Material | Method Used | Substrates | Electrodes | T_max,Process_ (°C) | d_33,eff_ (pm/V) | P_r_ (μC/cm^2^) | E_c_ (kV/cm) | Notes | Ref |
| --- | --- | --- | --- | --- | --- | --- | --- | --- | --- | --- | --- |
| Monroe  (This work) | **2022** | KNbO3 | SP | Silicon, Paper | Au (Evaporated) | 120 | 13 | - | - |  |  |
| Stojanovic | **2004** | BaTiO_3_ | SP | Al_2_O_3_ | Ag-Pd | 850 | - | 0.0107 | 0.00003 |  | ^1^ |
| Wagiran | **2005** | BaTiO_3_ | SP | Ceramic Glass | Ag (SP) | 850 | - | - | - | BTO acts as humidity sensor, no piezoelectric values reported | ^2^ |
| Chen | **2022** | BaTiO_3_ | DLP | - | Ag (SP) | 1300 | 146 | - | - |  | ^3^ |
| Wang | **2020** | BaTiO_3_ | SLP | - | Ag (SP) | 1320 | 190 | - | - |  | ^4^ |
| Sakai | **2006** | BaTiO_3_ | IJP | ZrO_2_ | Pt (SP) | 1400 (Pt) 600 (BTO) | - | 3.1 | 1.1 |  | ^5^ |
| Almeida | **2004** | BaTiO_3_ - HA | SP | Al_2_O_3_ | Ag (SP) | 850 | - | - | - | Values not reported | ^6^ |
| Lim | **2017** | BaTiO_3_-epoxy | IJP | PI | Ag (IJP) | 250  (+NIR oven) | - | - | - | Assumed d_33_ = 78 pC/N from COMSOL | ^7^ |
| Nguyen | **2022** | BaTiO_3_-PUA | SP | Steel | Ag (SP) | 150  (+UV Cure) | 1.31 | - | - |  | ^8^ |
| Liu | **2020** | Bi_0.5_Na_0.5_TiO_3_ | SP | MgO, Al_2_O_3_ | Pt (SP) / Au (SP) | 1380 (Pt) 900 (BNTO) | 80 / 50 | 10 | 35 |  | ^9^ |
| Markham | **2017** | Hydroxyapatite | SP | ITO | ITO (Film) | 25 | 0.02 | - | - |  | ^10^ |
| Korostynska | **2011** | Hydroxyapatite | SP | Al | Al (Foil) | 400 | - | - | - | Values not reported | ^11^ |
| Silva | **2005** | Hydroxyapatite | SP | Al_2_O_3_ | Ag (SP) | 700 | - | - | - | Values not reported | ^12^ |
| Badurova | **2020** | Hydroxyapatite | SP | ITO | ITO (Film) | 1200 | 0.03 | - | - |  | ^13^ |
| Zhang | **2010** | KNBT | SP | Al_2_O_3_ | Pt (SP) | 850 | 88 | 5-10 | 57.3 |  | ^14^ |
| Levassort | **2011** | KNN | SP, Pad Printing | - | Au / Ag (Printed) | 1000 | - | - | - | Values not reported | ^15^ |
| Li | **2012** | KNN | SP | Glass |  | 1100 | 133 | 20 | 9.1 |  | ^16^ |
| Pavlic | **2012** | KNN | SP | Al_2_O_3_ | Pt (SP) | 1100 | - | - | - | Values not reported | ^17^ |
| Pavlic | **2014** | KNN | SP | Al_2_O_3_ | Pt (SP) | 1100 | 44 | - | - |  | ^18,19^ |
| Hansen | **2009** | KNN-LT | SP | Al_2_O_3_ | ? (SP) | 1000 | 86 | - | - |  | ^20^ |
| Mercier | **2017** | KNN-Sr | SP | Al_2_O_3_ | Pt (SP) | 1200 | 80 | - | - |  | ^21,22^ |
| Hubler | **2012** | PVDF-TrFE | Flexo-graphic | Paper | PEDOT:PSS (Flexographic) | 130 | - | - | - | Values not reported | ^23^ |
| Haque | **2016** | PVDF-TrFE | IJP | PI, PET | Ag (IJP) | 140 | - | - | - | Values not reported | ^24^ |
| Sekine | **2016** | PVDF-TrFE | IJP | PEN | Ag (IJP) | 180 | - | 5 | 5 |  | ^25^ |
| Goncalves | **2019** | PVDF-TrFE | SP | Glass | Ag (SP) | 230 | 19 | - | - |  | ^26^ |
| Glinsek | **2022** | PZT | IJP | Glass | ITO | 700 | - | 4 | - | Reported e_33_* = 7.7 C/m^2^ | ^27^ |
| Glynne-Jones | **2000** | PZT | SP | Si | Pt (e-beam Evap) / Ag (SP) | 750 | 101 | - | - |  | ^28^ |
| Kok | **2009** | PZT | SP | Carbon paste (Sacrificial) | Ag-Pd (SP) | 800 | 53 | - | - |  | ^29^ |
| Chen | **1995** | PZT | SP | Si | Ti-Pt | 850 | 38 | 2.5 | 40 |  | ^30^ |
| Very | **2013** | PZT | SP | Al_2_O_3_ | Ag-Pd | 850 | 0.15 | - | - |  | ^31^ |
| Grall | **2020** | PZT | SP | Al_2_O_3_ | Au (SP) | 900 | - | - | - | Values not reported | ^32^ |
| Debeda | **2015** | PZT | SP | Al_2_O_3_ | Au (SP) | 920 | - | - | - | Values not reported | ^33^ |
| Zhu | **2000** | PZT | SP | Al_2_O_3_ | Ag-Pd | 1030 | 41 (d_31_) | - | - |  | ^34^ |
| Torah | **2004** | PZT | SP | Al_2_O_3_ | Ag-Pd | 750 | 33 | - | - |  | ^35^ |
| Sivanandan | **2008** | PZT | SP | Al_2_O_3_ | Pt (SP) | 1000 | - | - | - | Reported e_31_* = -4.00 C/m^2^ | ^36^ |
| Yildirim | **2020** | PZT-PDMS | SP  (Blade casting) | Glass, PET | ITO | - | - | - | - | Values not reported | ^37^ |
| Garcia-Farrera | **2019** | ZnO | EHD | Si, PET | ITO (Sputtered) | 25 | 23^(PFM)^ | - | - |  | ^38^ |
| Garcia | **2021** | ZnO | Gravure | PET | ITO | 100 | 4 | - | - | Printed only a seed layer and  grew the ZnO layer via CBD over 16 hr | ^39^ |

# SI.2. Particle Size Distribution Analysis

Samples of an ink containing the ground or unground KN particulate are fabricated by screen printing onto a silicon substrate. The samples are then cleaved in liquid nitrogen to produce a clean cross-section. Images are then taken using a scanning electron microscope of the cross section, with clearly resolved particulate. ImageJ processing software is then used to evaluate the average particle diameter from the images by first assigning randomly defined lines across an image and identifying the number of particulates occurring in that length of the cross section. This is repeated at least 50 times to achieve a distribution of particle sizes such that statistics can be completed with confidence. This entire process is repeated for both the ground and unground KN powders.


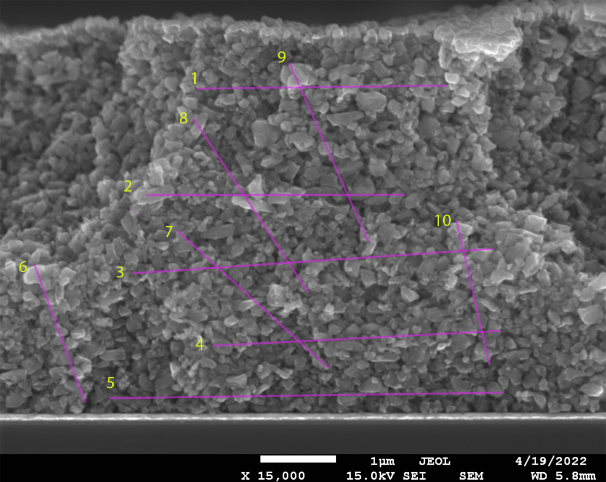

**Figure SI.2.1**. (a) An SEM micrograph of the printed KN layer post-grinding, with annotated lines used for particle sizing. (b) Particle size distribution for both the unground (orange) and ground (green) KNbO_3_ particles.

# SI.3. Device Design

## SI.3.1. Resonance Frequency Model

| $f_{r}=\frac{{1.875}^{2}}{2\pi L^{2}}\sqrt{\frac{\left\langle EI \right\rangle_{eq}}{\left\langle\rho A \right\rangle}}=\frac{{1.875}^{2}}{2\pi L^{2}}\sqrt{\frac{\left\langle EI \right\rangle_{eq}}{w_{c}\left( \rho_{sub}h_{sub}+ \rho_{piezo}h_{piezo} \right)}}$ | ( 3*.*1 ) |
| --- | --- |
| $\left\langle EI \right\rangle_{eq}=\frac{w_{c}\left[ E_{sub}^{2}h_{sub}^{4}+E_{piezo}^{2}h_{piezo}^{4}+E_{sub}E_{piezo}h_{sub}h_{piezo}\left( 4h_{sub}^{4}+6h_{sub}h_{piezo}+4h_{sub}^{4} \right) \right]}{12\left( E_{sub}h_{sub}+E_{piezo}h_{piezo} \right)}$ | ( 3.2 ) |

**Table SI.3.1**. Variables associated with resonance frequency model equations 3.1 and 3.2.

| **Variable** | **Units** | **Description** | **Values and Constraints** |
| --- | --- | --- | --- |
| $f_{r}$ | Hz | Cantilever resonance frequency | 1-20 kHz |
| $L$ | m | Length of the resonator | $>5*w_{c}$ |
| $w_{c}$ | m | Width of the resonator | $>0.8 mm$ |
| $h_{total}$ | m | Thickness of the resonator | $h_{piezo}+h_{sub}$ |
| $h_{sub}$ | m | Thickness of the substrate | $h_{Si}=247 um$  $h_{paper}=200 um$ |
| $h_{piezo}$ | m | Thickness of the piezoelectric | 0.01 mm |
| $E_{sub}$ | Pa | Young’s modulus of the substrate | $E_{Si}=150 GPa$  $E_{paper}\approx1.9 GPa$* |
| $E_{piezo}$ | Pa | Young’s modulus of the piezo | $E_{KN}\approx73 GPa$* |
| $\rho_{sub}$ | kg/m^3^ | Density of the substrate | $\rho_{Si}=2330 kg/m^{3}$  $\rho_{paper}\approx1025 kg/m^{3}$* |
| $\rho_{piezo}$ | kg/m^3^ | Density of the piezoelectric layer | $\rho_{KN}\approx2500 kg/m^{3}$* |

*  These values have been estimated, see section SI.3.3 for details.

## SI.3.2. Constraints and assumptions

**Assumption**: The thickness of the thermally evaporated electrodes is negligible compared to the thickness of the piezoelectric layer and substrate, permitting the assumption of a bimorph cantilever.

**Constraint on resonance frequency:** The sample must resonate in a frequency range easily detectable by available equipment, and this is limited to the range of 1-20 kHz.

## SI.3.3. Approximations

Initial development cycles were conducted prior to the empirical evaluation of several material properties in the model above. Later tests (see following sections) would provide concrete values for the densities of the paper and piezoelectric layer, yet for first tests, these values along with the young’s moduli of the paper and piezoelectric layer had to be approximated from literature values.

For the paper substrate, literature predictions were used at first to have a range for density and Young’s modulus values, which later were refined while doing FEM simulations. For the piezoelectric layer, a simplified composite approximation was initially used with literature predictions to determine a numerical range for these values. This assumes that the final ink layer will contain all components of the ink recipe except the solvent, which is evaporated upon drying the ink.

| $V_{binder}=\frac{m_{binder}}{\rho_{binder}}$ and $V_{active}=\frac{m_{active}}{\rho_{active}}$ | ( 3.3 ) |
| --- | --- |
| $f=\frac{V_{binder}}{V_{binder}+V_{active}}$ | ( 3.4 ) |
| $E_{ink,high}=f*E_{binder}+\left( 1-f \right)*E_{active}$ | ( 3.5 ) |
| $E_{ink,low}=\frac{1}{\left( \frac{f}{E_{binder}}+\frac{(1-f)}{E_{active}} \right)}$ | ( 3.6 ) |
| $\rho_{ink,high}=f*\rho_{binder}+\left( 1-f \right)*\rho_{active}$ | ( 3.7 ) |
| $\rho_{ink,low}=\frac{1}{\left( \frac{f}{\rho_{binder}}+\frac{(1-f)}{\rho_{active}} \right)}$ | ( 3.8 ) |

**Table SI.3.2**. Variables associated with material property approximation model equations 3.3 – 3.8.

| **Variable** | **Units** | **Description** | **Values and Constraints** |
| --- | --- | --- | --- |
| $m_{binder}$ | $g$ | Specific mass of the binding ingredient (ethyl cellulose) | 11.5 g  (Defined by Ink optimization) |
| $\rho_{binder}$ | $g/cm^{3}$ | Density of the binding ingredient (ethyl cellulose) | 1.05 g/cm^3^  (from Literature) |
| $m_{active}$ | $g$ | Specific mass of the active ingredient (KNbO_3_) | 88.5 g  (Defined by Ink optimization) |
| $\rho_{active}$ | $g/cm^{3}$ | Density of the active ingredient (KNbO_3_) | 4.37 g/cm^3^  (from Literature) |
| $E_{binder}$ | $GPa$ | Young’s Modulus of the binding ingredient (ethyl cellulose) | 2 GPa  (from Literature) |
| $E_{active}$ | $GPa$ | Young’s Modulus of the active ingredient (KNbO_3_) | 240-250 GPa  (from Literature) |
| $V_{binder}$ | $cm^{3}$ | Specific volume of the binding ingredient (ethyl cellulose) | 10.97 $cm^{3}$  (Calculated) |
| $V_{active}$ | $cm^{3}$ | Specific volume of the active ingredient (KNbO_3_) | 20.25 $cm^{3}$  (Calculated) |
| $f$ | $[-]$ | Filler ratio | 0.35  (Calculated) |
| $E_{ink,high}$ | $GPa$ | Estimated Young’s Modulus of the composite ink, high value | 156.4 GPa  (Calculated) |
| $E_{ink,low}$ | $GPa$ | Estimated Young’s Modulus of the composite ink, low value | 5.6 GPa  (Calculated) |
| $\rho_{ink,high}$ | $g/cm^{3}$ | Estimated density of the composite ink, high value | $3.2 g/cm^{3}$  (Calculated) |
| $\rho_{ink,low}$ | $g/cm^{3}$ | Estimated density of the composite ink, low value | $2.1 g/cm^{3}$  (Calculated) |


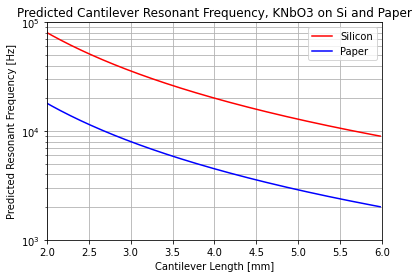


**Figure SI.3.1.** Predicted resonance frequency of the cantilevers as a function of length for both substrate materials (Si and paper).

# SI.4. Cantilever Device Dimensions

**
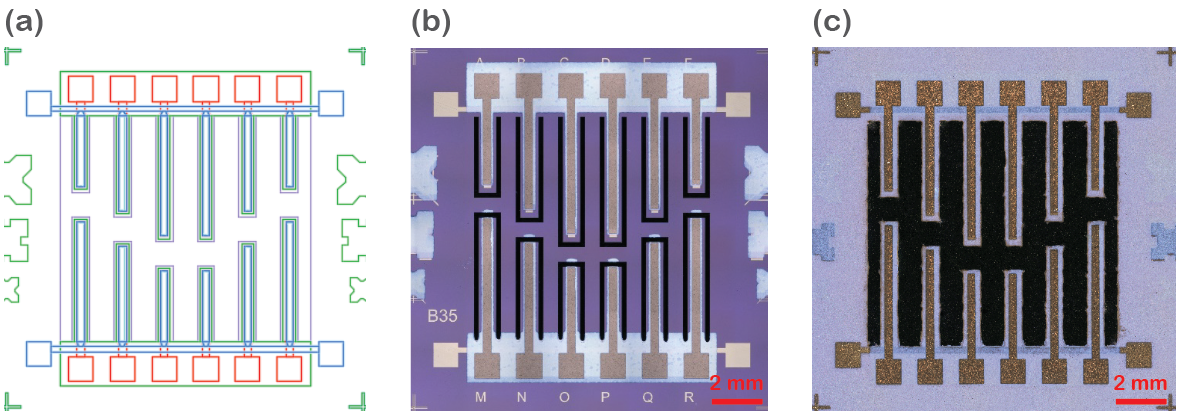
**

**Figure SI.4.1.** (a) CAD design of cantilever devices. (b) Fully printed cantilever devices on silicon. (c) Fully printed cantilever devices on paper.

**Table SI.4.1.** Target dimension values for all components in the cantilever devices.

|  | **Units** | **Si Substrate** | **Paper Substrate** | **Piezoelectric** | **Electrodes** |
| --- | --- | --- | --- | --- | --- |
| **Thickness,** $\boldsymbol{h}$ | μm | 247 | 200-230 | 8.4-11.2 | 0.11 |
| **Length,** $\boldsymbol{L}$ | mm | 3.1, 4.1, 5.1 | | 3.0, 4.0, 5.0 | 2.9, 3.9, 4.9 |
| **Width,** $\boldsymbol{w}$ | mm | 0.7 | | 0.5 | 0.3 |

# SI.5. Material Density Calculations


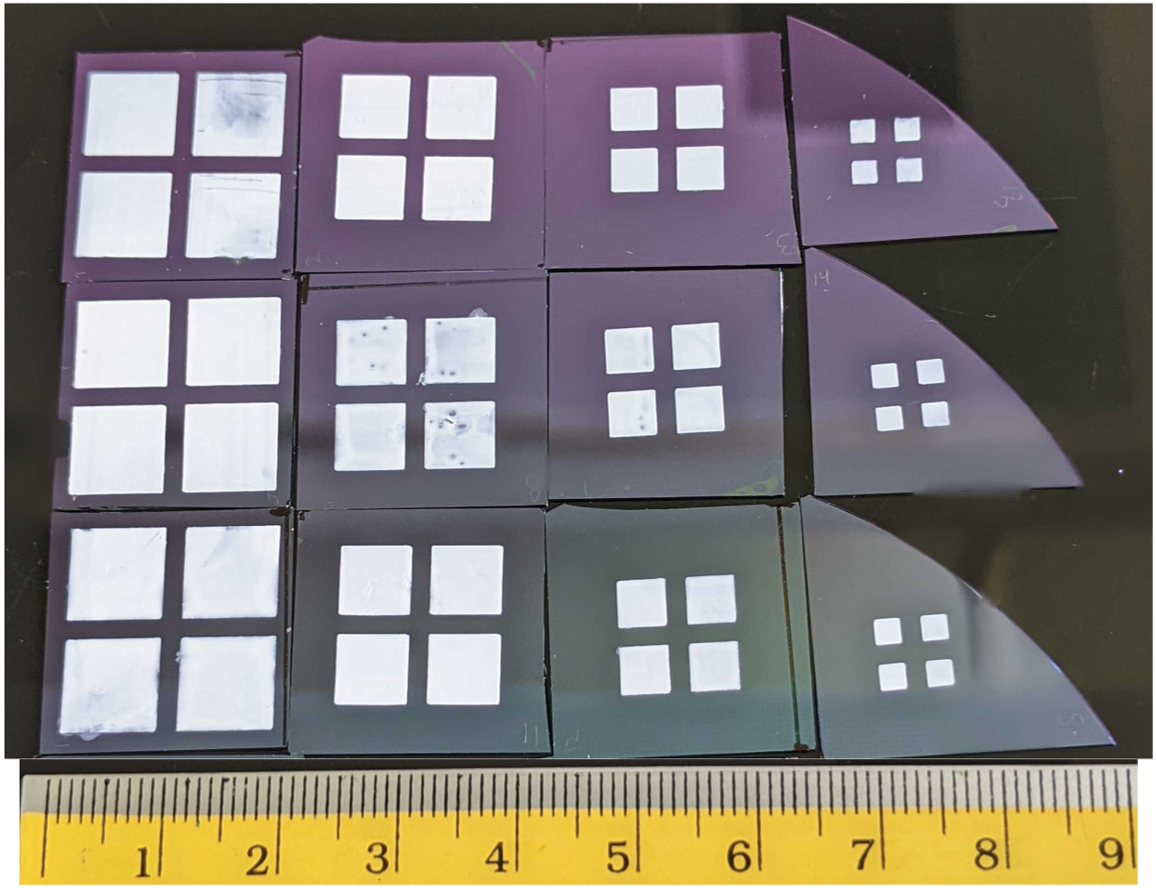

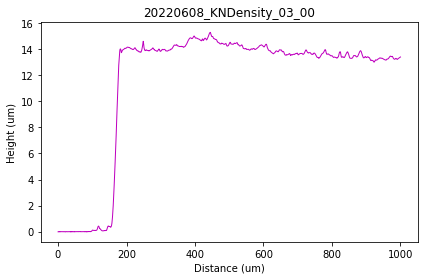


**Figure SI.5.1.** (a) Printed KN Ink samples for density calculations, printed on a 100 mm diameter silicon wafer. (b) Sample profilometry data for samples printed above. Such data used to determine printed film thickness as a means of determining sample printed volume.

**Figure SI.5.2.** Mass as a function of volume, as used for determination of density for (a) Printed KNbO_3_ ink layer and (b) Paper substrates.

# SI.6. Ink Rheology

**Figure SI.6.1.** Rheometer data for KN screen printing ink as measured at room temperature.

# SI.7. Ink Adhesion


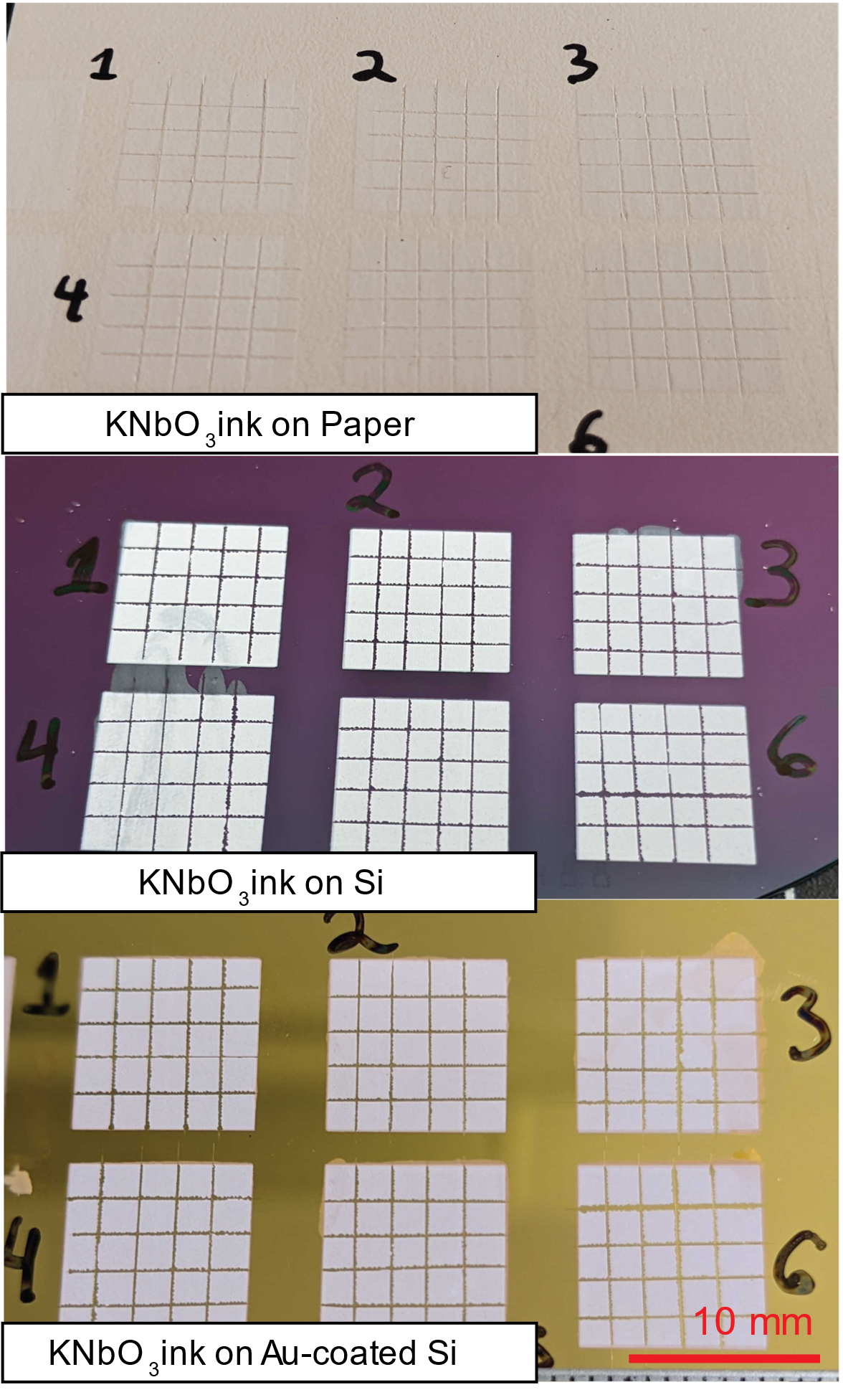


**Figure SI.7.1.** Samples for adhesion test on three different substrates (paper, Si, and gold-coated Si), showing nice adhesion, with no major difference in adhesion between the three tested substrates. Adhesion tests were conducted on the printed layers following ASTM 1842-15.

# SI.8. Berlincourt Measurement Methodology

A Berlincourt measurement is used to evaluate the effective piezoelectric coefficient, $d_{33,eff}$, by simultaneously applying a force to the sample and a reference piezoelectric material of known properties. Since the two piezoelectrics (the reference and the Device under test) are mechanically coupled and thus experience the same applied force, the piezoelectric coefficient of the device under test can be determined by looking at the electrical response of the sample as compared to the reference piezoelectric.

| $D=d*\sigma=d_{33}\left( \frac{F_{appl}}{A} \right)$ | ( 8.1 ) |
| --- | --- |
| $D*A=d_{33}*F_{appl}= Q_{free}=CV$ | ( 8.2 ) |
| $V=d_{33}\left( \frac{F_{appl}}{C} \right)$ | ( 8.3 ) |
| $F_{appl,DUT}=F_{appl,Ref}$ | ( 8.4 ) |
| $C_{DUT}=C_{Ref}$ | ( 8.5 ) |
| $\frac{V_{DUT}}{d_{33,DUT}}=\frac{V_{Ref}}{d_{33,Ref}}$ | ( 8.6 ) |
| $\boldsymbol{d}_{\boldsymbol{33,DUT}}\boldsymbol{=}\boldsymbol{d}_{\boldsymbol{33,Ref}}\left( \frac{\boldsymbol{V}_{\boldsymbol{DUT}}}{\boldsymbol{V}_{\boldsymbol{Ref}}} \right)\left[ \mathbf{=} \right]\mathbf{pC/N}$ | ( 8.7 ) |

**Table SI.8.1**. Variables associated with Berlincourt method equations 8.1 – 8.7.

| **Variable** | **Units** | **Description** |
| --- | --- | --- |
| $D$ | $C/m^{2}$ | Electric displacement field |
| $d$ | $pC/N$ | Piezoelectric tensor |
| $\sigma$ | ${N/m}^{2}$ | Stress tensor |
| $F_{appl}$ | $N$ | Force applied to both piezoelectric devices |
| $A$ | $m^{2}$ | Area across which the force is applied |
| $Q_{free}$ | $C$ | Charge held on the conductors |
| $C$ | $F$ | Capacitance |
| $V$ | $V$ | Electric potential |


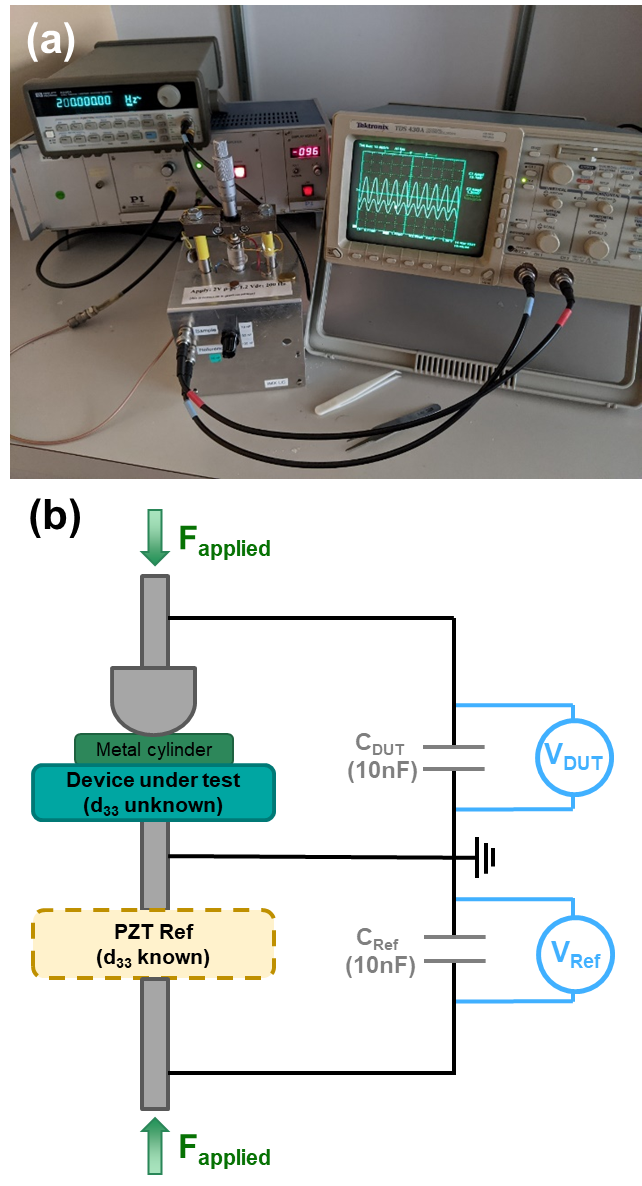


**Figure SI.8.1.** (a) Image of Berlincourt measurement setup. (b) Diagram of simplified Berlincourt setup circuit.

# SI.9. LDV-based d_31_ Calculations

First the output signal of the LDV is converted from a voltage to a displacement value (Eq. 9.1), then, a Lorentzian fit is used to decouple the displacement at the cantilever time from the quality factor of the resonator.

| $u_{LDV}\left( \omega\right)=Responsivity\left( \frac{V_{LDV}}{\omega} \right)$ | ( 9.1 ) |
| --- | --- |
| $u_{n,emp}\left( \omega\right)\approx\chi_{n}^{A}\left( \frac{d_{31,emp}z_{offset}L^{2}}{h_{total}^{3}} \right)\left( \frac{w_{elec}}{w_{c}} \right)\left( \frac{E_{piezo}}{E_{sub}} \right)\left( \frac{V_{act}}{1-\left( \frac{\omega}{\omega_{n}} \right)^{2}+j\frac{\omega}{\omega_{n}Q}} \right)$ | ( 9.2 ) |

With this, we extract the corresponding static displacement when actuated using 1V, $u_{static,1V}$ . We compare this to the deflection of COMSOL-modelled cantilevers with the same material properties and dimensions. The only tuning parameter we used was the $d_{31,model}$ until the model matched the empirical values extracted in Eq. 9.2.

**Table SI.9.1**. Variables associated with Laser Doppler Vibrometry data analysis equations 9.1 – 9.5.

| **Variable** | **Units** | **Description** |
| --- | --- | --- |
| $u(\omega)$ | m | Measured deflection of the cantilever tip, as a function of angular velocity |
| $u_{LDV}$ | m | Measured deflection of the cantilever tip |
| $u_{n}$ | m | Maximum deflection at the cantilever tip at the resonance frequency |
| $\omega$ | $1/s$ | Frequency of the applied signal |
| $\omega_{n}$ | $1/s$ | Resonance frequency of the cantilever |
| $f=\frac{\omega}{2\pi}$ | Hz | Frequency of the applied signal |
| $\mathrm{Responsivity}$ | $\left( \frac{mm}{s} \right)/V$ | Responsivity of the LDV velocity decoder |
| $V_{act}$ | $V$ | Voltage of the applied actuating signal |
| $V_{LDV}$ | $V$ | Output signal of the LDV velocity measurement |
| $\chi_{n}^{A}$ | $[-]$ | Proportionality parameter,  approximated as 1.38 |
| $L$ | $m$ | Length of the cantilever |
| $h_{total}$ | $m$ | Thickness of the resonator |
| $w_{c}$ | $m$ | Width of the resonator |
| $w_{elec}$ | $m$ | Width of the electrode |
| $z_{0}$ | $m$ | Neutral axis (the plane in *z* with no longitudinal strain) |
| $z_{offset}=h_{total}-z_{0}$ | $m$ | Off-axis placement of the piezoelectric layer with respect to the neutral axis |
| $Q$ | $[-]$ | Quality factor of the cantilever |
| $E_{piezo}$ | Pa | Young’s modulus of the piezoelectric layer |
| $E_{Sub}$ | Pa | Young’s modulus of the substrate layer |

# SI.10. Bibliography for supplemental information

1. Stojanovic, B. D., Foschini, C. R., Pejovic, V. Z., Pavlovic, V. B. & Varela, J. A. Electrical properties of screen printed BaTiO3 thick films. *J Eur Ceram Soc* **24**, 1467–1471 (2004).

2. Wagiran, R. *et al.* Characterization of screen printed BaTiO3 thick film humidity sensor. *Int. J. Engineer. Techn.* **2**, 22–26 (2005).

3. Chen, X. *et al.* Effect of the particle size on the performance of BaTiO3 piezoelectric ceramics produced by additive manufacturing. *Ceram Int* **48**, 1285–1292 (2022).

4. Wang, W. *et al.* Fabrication of piezoelectric nano-ceramics via stereolithography of low viscous and non-aqueous suspensions. *J Eur Ceram Soc* **40**, 682–688 (2020).

5. Sakai, Y., Futakuchi, T. & Adachi, M. Preparation of BaTiO 3 Thick Films by Inkjet Printing on Oxygen-Plasma-Modified Substrates. *Jpn J Appl Phys* **45**, 7247–7251 (2006).

6. Almeida, A. F. L. *et al.* Optical and electrical properties of barium titanate-hydroxyapatite composite screen-printed thick films. *Solid State Sci* **6**, 267–278 (2004).

7. Lim, J. *et al.* All-inkjet-printed flexible piezoelectric generator made of solvent evaporation assisted BaTiO3 hybrid material. *Nano Energy* **41**, 337–343 (2017).

8. Nguyen, V.-C. *et al.* Printing smart coating of piezoelectric composite for application in condition monitoring of bearings. *Mater Des* **215**, 110529 (2022).

9. Liu, L., Karaki, T., Fujii, T. & Sakai, Y. Effect of substrate material to the properties of screen-printed lead free (Bi0.5Na0.5)TiO3-based thick films. *Jpn J Appl Phys* **59**, 0–6 (2020).

10. Markham, S. K., Stapleton, A., Haq, E. U., Kowal, K. & Tofail, S. A. M. Piezoelectricity in screen-printed hydroxyapatite thick films. *Ferroelectrics* **509**, 99–104 (2017).

11. Korostynska, O., Gigilashvili, G., Gandhi, A. A. & Tofail, S. A. M. High temperature induced pyroelectricity in screen-printed Hydroxyapatite thick films. in *14th International Symposium on Electrets* 141–142 (IEEE, 2011). doi:10.1109/ISE.2011.6085022.

12. Silva, C. C. *et al.* Hydroxyapatite screen-printed thick films: optical and electrical properties. *Mater Chem Phys* **92**, 260–268 (2005).

13. Badurova, K. *et al.* Piezoelectricity in Sr doped thick films of hydroxyapatite. *IEEE Transactions on Dielectrics and Electrical Insulation* **27**, 1409–1414 (2020).

14. Zhang, H., Jiang, S., Xiao, J. & Kajiyoshi, K. Low temperature preparation and electrical properties of sodium-potassium bismuth titanate lead-free piezoelectric thick films by screen printing. *J Eur Ceram Soc* **30**, 3157–3165 (2010).

15. Levassort, F. *et al.* High frequency single element transducer based on pad-printed lead-free piezoelectric thick films. in *2011 IEEE International Ultrasonics Symposium* 848–851 (IEEE, 2011). doi:10.1109/ULTSYM.2011.0207.

16. Li, Y., Hui, C., Wu, M., Li, Y. & Wang, Y. Textured (K0.5Na0.5)NbO3 ceramics prepared by screen-printing multilayer grain growth technique. *Ceram Int* **38**, S283–S286 (2012).

17. Pavlič, J., Kosec, M., Holc, J. & Rojac, T. K0.5Na0.5NbO3 thick films: preparation and properties. (2012).

18. Pavlič, J., Malič, B. & Rojac, T. Small Reduction of the Piezoelectric d 33 Response in Potassium Sodium Niobate Thick Films. *Journal of the American Ceramic Society* **97**, 1497–1503 (2014).

19. Pavlič, J., Malič, B. & Rojac, T. Microstructural, structural, dielectric and piezoelectric properties of potassium sodium niobate thick films. *J Eur Ceram Soc* **34**, 285–295 (2014).

20. Hansen, K., Astafiev, K. & Zawada, T. Lead-free piezoelectric thick films based on potassium sodium niobate solutions. *Proc IEEE Ultrason Symp* 1738–1741 (2009) doi:10.1109/ULTSYM.2009.5441677.

21. Mercier, H., Kuscer, D. & Levassort, F. Processing and sintering of sodium-potasium niobate–based thick films. *Journal of Microelectronics, Electronic Components and Materials* **47**, 179–185 (2017).

22. Mercier, H. *et al.* Electrophoretic deposition and properties of strontium-doped sodium potassium niobate thick films. *J Eur Ceram Soc* **37**, 5305–5313 (2017).

23. Hübler, A. C. *et al.* Fully mass printed loudspeakers on paper. *Org Electron* **13**, 2290–2295 (2012).

24. Haque, R. I. *et al.* Inkjet printing of high molecular weight PVDF-TrFE for flexible electronics. *Flexible and Printed Electronics* **1**, 015001 (2016).

25. Sekine, T. *et al.* Fully printed and flexible ferroelectric capacitors based on a ferroelectric polymer for pressure detection. *Jpn J Appl Phys* **55**, 10TA18 (2016).

26. Gonçalves, S. *et al.* Environmentally Friendly Printable Piezoelectric Inks and Their Application in the Development of All-Printed Touch Screens. *ACS Appl Electron Mater* **1**, 1678–1687 (2019).

27. Glinsek, S. *et al.* Inkjet‐Printed Piezoelectric Thin Films for Transparent Haptics. *Adv Mater Technol* **7**, 2200147 (2022).

28. Glynne-Jones, P., Beeby, S. P., Dargie, P., Papakostas, T. & White, N. M. An investigation into the effect of modified firing profiles on the piezoelectric properties of thick-film PZT layers on silicon. *Meas Sci Technol* **11**, 526–531 (2000).

29. Kok, S.-L., White, N. M. & Harris, N. R. Fabrication and characterization of free-standing thick-film piezoelectric cantilevers for energy harvesting. *Meas Sci Technol* **20**, 124010 (2009).

30. Chen, H. D., Udayakumar, K. R., Cross, L. E., Bernstein, J. J. & Niles, L. C. Dielectric, ferroelectric, and piezoelectric properties of lead zirconate titanate thick films on silicon substrates. *J Appl Phys* **77**, 3349–3353 (1995).

31. Very, F. *et al.* Piezoelectric thick film sensors: Fabrication and characterization. in *2013 Joint IEEE International Symposium on Applications of Ferroelectric and Workshop on Piezoresponse Force Microscopy (ISAF/PFM)* 287–290 (IEEE, 2013). doi:10.1109/ISAF.2013.6748690.

32. Grall, S., Santawitee, O., Dufour, I., Aubry, V. & Debéda, H. New corn-based sacrificial layer for MEMS based on screen-printed PZT ceramics. *Sens Actuators A Phys* **304**, 111826 (2020).

33. Debéda, H., Clément, P., Llobet, E. & Lucat, C. One-step firing for electroded PZT thick films applied to MEMS. *Smart Mater Struct* **24**, (2015).

34. Zhu, W., Yao, K. & Zhang, Z. Design and fabrication of a novel piezoelectric multilayer actuator by thick-film screen printing technology. *Sens Actuators A Phys* **86**, 149–153 (2000).

35. Torah, R. N., Beeby, S. P. & White, N. M. Improving the piezoelectric properties of thick-film PZT: The influence of paste composition, powder milling process and electrode material. *Sens Actuators A Phys* **110**, 378–384 (2004).

36. Sivanandan, K., Achuthan, A. T., Kumar, V. & Kanno, I. Fabrication and transverse piezoelectric characteristics of PZT thick-film actuators on alumina substrates. *Sens Actuators A Phys* **148**, 134–137 (2008).

37. Yildirim, A. *et al.* Roll‐to‐Roll Production of Novel Large‐Area Piezoelectric Films for Transparent, Flexible, and Wearable Fabric Loudspeakers. *Adv Mater Technol* **5**, 2000296 (2020).

38. García-Farrera, B. & Velásquez-García, L. F. Ultrathin Ceramic Piezoelectric Films via Room-Temperature Electrospray Deposition of ZnO Nanoparticles for Printed GHz Devices. *ACS Appl Mater Interfaces* **11**, 29167–29176 (2019).

39. Garcia, A. J. L. *et al.* Low-Temperature Growth of ZnO Nanowires from Gravure-Printed ZnO Nanoparticle Seed Layers for Flexible Piezoelectric Devices. *Nanomaterials* **11**, 1430 (2021).
